# Supplementary material for: Proteomics of CKD progression in the chronic renal insufficiency cohort
Source: Nat Commun. 2023 Oct 10;14:6340. doi: 10.1038/s41467-023-41642-7 (PMC10564759; doi:10.1038/s41467-023-41642-7)
Supplement: Supplementary file 1 — Supplementary Information [file 41467_2023_41642_MOESM1_ESM.pdf]

Supplementary Figure 1

Hazard Ratios For CKD Progression in Participants With and Without Diabetes

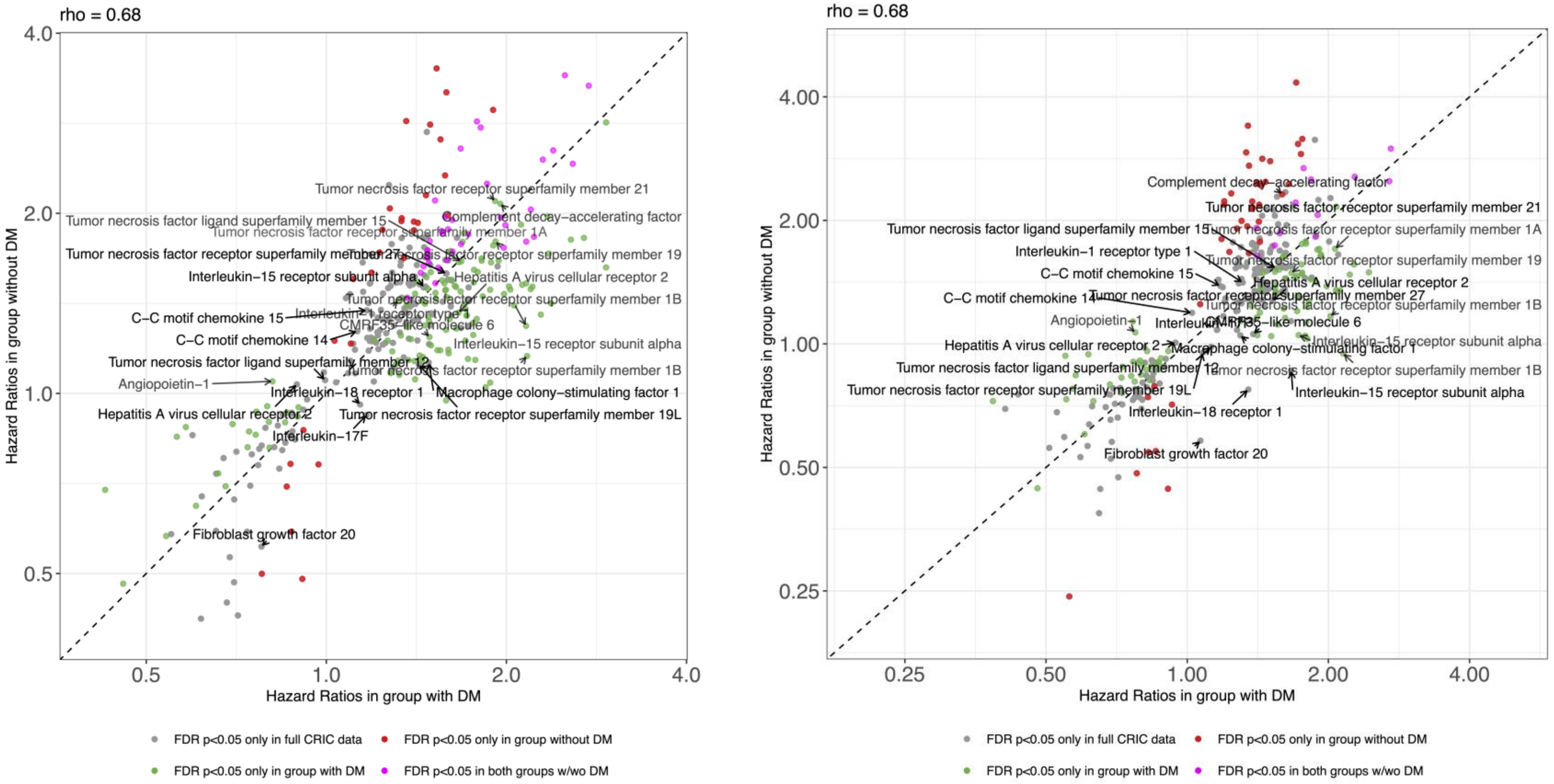

Fully adjusted log2 hazard ratios for the outcome 10-year ESRD/50% eGFR decline (left) and ESRD alone (right), shown in participants with or without DM, for each of the proteins that were associated with respective outcomes in the full CRIC cohort at FDR < 0.05 (330 proteins for 10-year ESRD/50% eGFR decline and 287 for ESRD alone). Fully adjusted model includes age, gender, race, systolic blood pressure, tobacco use, body mass index, history of cardiovascular disease, eGFR, UPCR. Dotted line illustrates rho of 1.

Supplementary Figure 2

Calibration of the Proteomic Risk Models for Primary and Secondary Outcome in CRIC

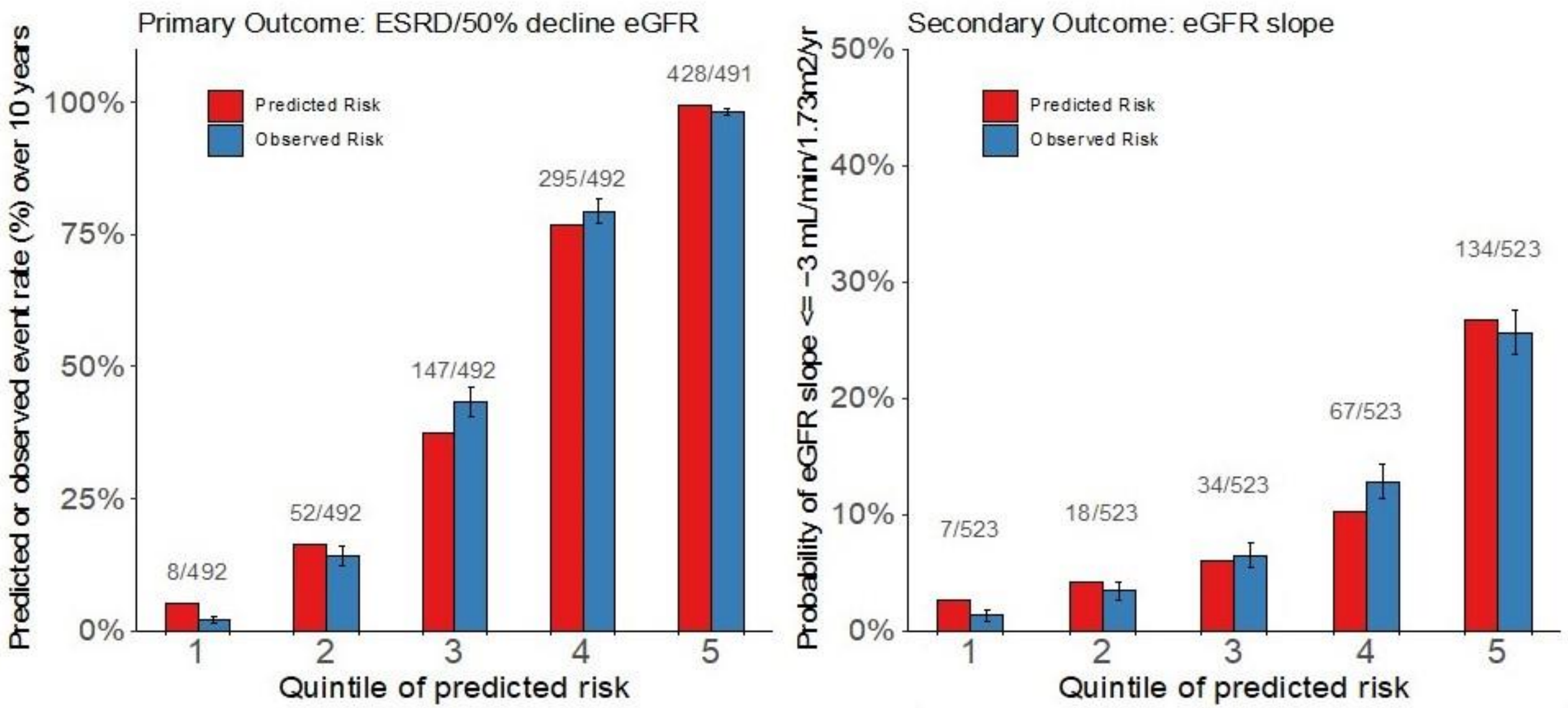

Model-based calibration p-value	Q1	Q2	Q3	Q4	Q5
	0.0080	0.26	0.14	0.12	0.31

Q1	Q2	Q3	Q4	Q5
0.0072	0.44	0.64	0.046	0.54

Calibration was performed in the training set, N=2459 for primary outcome, N=2615 for secondary outcome. Bars represents 1 standard error of observed risks of the primary and secondary outcomes in each quintile, respectively. The plots also illustrate the breadth of the dynamic range of the predicted and associated observed risk. For the primary outcome and secondary outcome, Q5/Q1 risk is 20 and 10, respectively. Calibration p-values are one-sided.

Supplementary Figure 3

Subgroup Analyses for KFRE and 65-Protein Risk Model

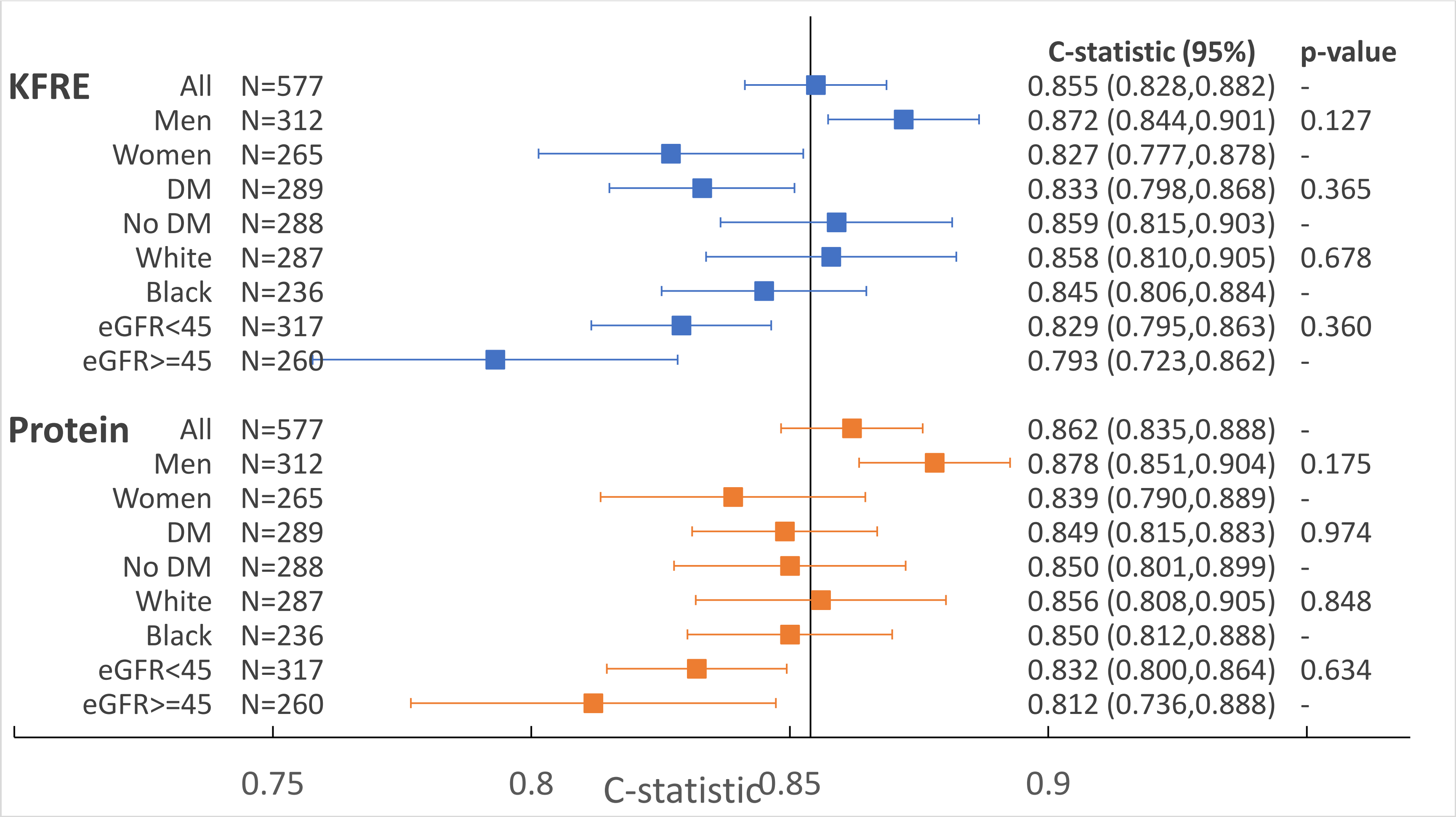

Point estimates and standard errors for c-statistic in CRIC for the primary renal outcome are shown in subgroups of gender, diabetes, race and eGFR. All tests use two-sided, raw p-values.

Supplementary Figure 4

Time-dependent AUCs For Risk Models of 10-Year CKD Progression

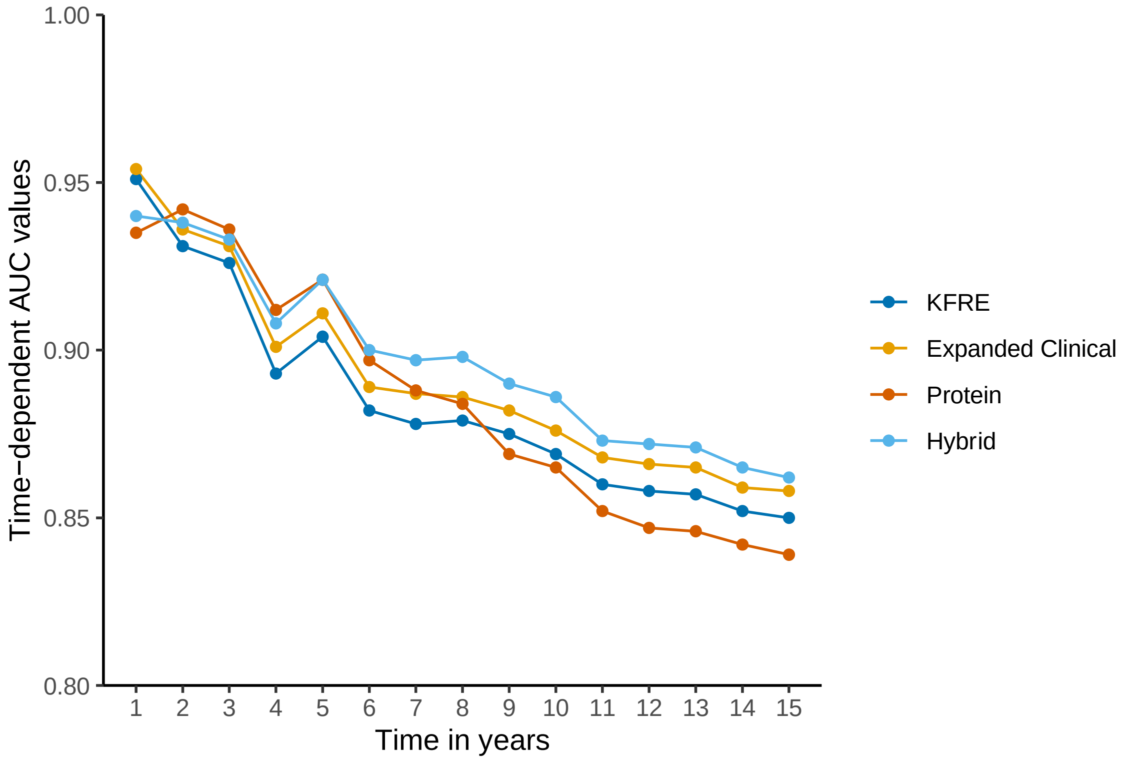

Time-dependent AUCs are shown for each risk model in the 20% testing set in CRIC.

The Chronic Renal Insufficiency Cohort (CRIC) Study Investigators

- Laura M. Dember, MD
  - Perelman School of Medicine at The University of Pennsylvania Scientific & Data Coordinating Center
- Debbie L Cohen, MD
  - University of Pennsylvania
- Lawrence Appel, MD, MPH
  - Johns Hopkins University ProHealth
- Jeffrey Fink, MD, MS
  - University of Maryland
- Mahboob Rahman, MD, MS
  - University Hospitals Case Medical Center
- Edward J. Horwitz, MD
  - MetroHealth Medical Center
- Jonathan J. Taliercio, DO
  - The Cleveland Clinic Foundation
- Panduranga Rao, MD
  - University of Michigan Hospital and Health Systems
- James H. Sondheimer, MD
  - Wayne State University School of Medicine
- James P. Lash, MD
  - University of Illinois at Chicago
- Jing Chen, MD, MMSc, MSc
  - Tulane Office of Health Research
- Alan S. Go, MD
  - Kaiser Permanente of Northern California
- Chi-yuan Hsu, MD, MSc
  - University of California, San Francisco
- Vallabh O Shah, PhD, MS,
  - University of New Mexico Health Sciences Center
- Mark L. Unruh, MD, MS
  - University of New Mexico Health Sciences Center
- Robert G. Nelson, MD, PhD, MS
  - National Institute of Diabetes and Digestive and Kidney Diseases (NIDDK) | National Institutes of Health (NIH)
- Amanda Anderson PhD, MPH
  - Tulane Office of Health Research

CKD Biomarkers Consortium Collaborators: Phase II

**Boston University School of Medicine**	Vasan S. Ramachandran, M.D. (Chair, Steering Committee), Joseph Massaro Ph.D.
**Broad Institute**	Clary Clish Ph.D.
**Case Western Reserve University**	Jeffrey Schelling, M.D. (PI),
**The Children's Hospital of Philadelphia**	Michelle Denburg, M.D., MSCE (PI), Susan Furth, M.D., Ph.D.(PI)
**Children's Mercy Hospital**	Bradley Warady, M.D.
**Brigham and Women's Hospital, Harvard University**	Joseph Bonventre, M.D., Ph.D. (PI), Sushrut Waikar, M.D., MPH. (PI), Gearoid McMahon M.B., BCH, Venkata Sabbisetti Ph.D.
**Johns Hopkins University**	Josef Coresh, M.D., Ph.D. (PI), Morgan Grams M.D., Casey Rebholz, Ph.D., Alison Abraham, Ph.D., Adriene Tin Ph.D., Chirag Parikh, MD, PhD (PI)
**University of Louisville**	Jon Klein, M.D., Ph.D.
**Icahn School of Medicine at Mount Sinai**	Steven Coca, DO, MS (PI), Bart S Ferket, M.D., PhD, Girish N. Nadkarni, MD, MPH, CPH,
**Massachusetts General Hospital**	Eugene Rhee M.D. (PI)
**National Institutions of Health**	Paul L. Kimmel, M.D. (NIDDK), Daniel Gossett, Ph.D. (NIDDK)
**Ohio State University**	Brad Rovin, M.D. (PI)
**San Francisco VA Medical Center**	Michael G. Shlipak, M.D. (PI)
**Tufts Medical Center**	M Sarnak, M.D. (PI), Andrew S. Levey, M.D., Lesley A. Inker, M.D., M.S., Meredith Foster, Ph.D.
**University of Alabama at Birmingham**	Orlando M. Gutiérrez, M.D. (PI)
**University of California San Diego**	Joachim Ix, M.D. (PI)
**University of Texas Southwestern Medical Center**	Ruth Dubin, M.D. (PI)
**University of Minnesota**	Jesse Seegmiller, Ph.D.
**University of North Carolina**	Tom Hostetter, M.D. (PI),
**University of Pennsylvania**	Rajat Deo, M.D. (PI), Hongzhe Li, Ph.D., Yue Ren, M.S.
**University of Pennsylvania Coordinating Center**	Sarah J. Schrauben, M.D., MSCE (PI), Harold I. Feldman, M.D., MSCE, Amanda Anderson, PhD, Theodore Mifflin, Ph.D.,

	D.A.B.C.C., Dawei Xie, Ph.D., Haochang Shou Ph.D., Shawn Ballard, M.S., Krista Whitehead, M.S., Heather Collins, Ph.D.
**Yale School of Medicine**	Jason Greenberg, M.D.
**University of California, San Francisco**	Peter Ganz, M.D. (PI)
