# Supplementary material for: Proteomics of CKD progression in the chronic renal insufficiency cohort
Source: Nat Commun. 2023 Oct 10;14:6340. doi: 10.1038/s41467-023-41642-7 (PMC10564759; doi:10.1038/s41467-023-41642-7)
Supplement: Supplementary file 3 — Description of Additional Supplementary Files [file 41467_2023_41642_MOESM3_ESM.pdf]

Description of Additional Supplementary Files

Title: Supplementary Data 1

Characteristics of CRIC Participants

Title: Supplementary Data 2

ARIC Baseline Characteristics and Calibration

Title: Supplementary Data 3

Twenty Proteins With the Highest HR's for the Primary Outcome in CRIC and ARIC. Hazard ratios are per MAD unit. Biological functions and available drugs.

Title: Supplementary Data 4

Twenty Proteins With the Lowest HR's for the Primary Outcome in CRIC and ARIC. Hazard ratios are per MAD unit. Biological functions and available drugs.

Title: Supplementary Data 5

Twenty Proteins With the Highest HR's for the Primary Outcome in CRIC with Multivariable Adjustment. Hazard ratios are shown per log2 and per MAD unit.

Title: Supplementary Data 6

Twenty Proteins With the Lowest HR's for the Primary Outcome in CRIC with Multivariable Adjustment. Hazard ratios are shown per log2 and per MAD unit.

Title: Supplementary Data 7

Mendelian Randomization

Title: Supplementary Data 8

Twenty Proteins Analyzed in CRIC Participants With and Without Diabetes

Title: Supplementary Data 9

Twenty Proteins Associated with Higher Risk 4-year eGFR Decline

Title: Supplementary Data 10

Twenty Proteins Associated With Lower Risk of 4-Year eGFR Decline

Title: Supplementary Data 11

65 Proteins in the 10-year Risk Model for the Primary Outcome of ESRD/50% eGFR Decline

Title: Supplementary Data 12

20 Proteins in the Risk Model for 4-Year eGFR Decline

Title: Supplementary Data 13

Risk Models Derived for Different Time Horizons

Title: Supplementary Data 14

Discrimination of Refit KFRE and Protein Models Using Race-Free eGFR for Study Outcomes

Title: Supplementary Data 15

Aptamers in Somascan V 4.0

Title: Supplementary Data 16

Proteins Excluded From the Analyses
