# Supplementary material for: Proteomics of CKD progression in the chronic renal insufficiency cohort
Source: Nat Commun. 2023 Oct 10;14:6340. doi: 10.1038/s41467-023-41642-7 (PMC10564759; doi:10.1038/s41467-023-41642-7)
Supplement: Supplementary file 5 — Reporting Summary [file 41467_2023_41642_MOESM5_ESM.pdf]

nature portfolio

Corresponding author(s): Ruth Dubin

Last updated by author(s): 2023 08 20

Reporting Summary

Nature Portfolio wishes to improve the reproducibility of the work that we publish. This form provides structure for consistency and transparency in reporting. For further information on Nature Portfolio policies, see our Editorial Policies and the Editorial Policy Checklist.

Statistics

For all statistical analyses, confirm that the following items are present in the figure legend, table legend, main text, or Methods section.

n/a	Confirmed	
[ ]	[x]	The exact sample size (*n*) for each experimental group/condition, given as a discrete number and unit of measurement
[ ]	[x]	A statement on whether measurements were taken from distinct samples or whether the same sample was measured repeatedly
[ ]	[x]	The statistical test(s) used AND whether they are one- or two-sided<br>*Only common tests should be described solely by name; describe more complex techniques in the Methods section.*
[ ]	[x]	A description of all covariates tested
[ ]	[x]	A description of any assumptions or corrections, such as tests of normality and adjustment for multiple comparisons
[ ]	[x]	A full description of the statistical parameters including central tendency (e.g. means) or other basic estimates (e.g. regression coefficient) AND variation (e.g. standard deviation) or associated estimates of uncertainty (e.g. confidence intervals)
[ ]	[x]	For null hypothesis testing, the test statistic (e.g. *F*, *t*, *r*) with confidence intervals, effect sizes, degrees of freedom and *P* value noted<br>*Give P values as exact values whenever suitable.*
[x]	[ ]	For Bayesian analysis, information on the choice of priors and Markov chain Monte Carlo settings
[x]	[ ]	For hierarchical and complex designs, identification of the appropriate level for tests and full reporting of outcomes
[x]	[ ]	Estimates of effect sizes (e.g. Cohen's *d*, Pearson's *r*), indicating how they were calculated

Our web collection on statistics for biologists contains articles on many of the points above.

Software and code

Policy information about availability of computer code

Data collection	No software was used in data collection
Data analysis	R code packages that were used include R, version 4.0.3 (RStudio, Inc., Boston, MA. URL http://www.rstudio.com/), with the packages of glmnet (version 4.0-2), survival (version 3.2-7 pec (version 2019.11), compareC (version 1.3.1), forestplot (version 1.10), lme4 (version 1.1-26). These are listed on page 13 of Methods. There are no custom codes developed.

For manuscripts utilizing custom algorithms or software that are central to the research but not yet described in published literature, software must be made available to editors and reviewers. We strongly encourage code deposition in a community repository (e.g. GitHub). See the Nature Portfolio guidelines for submitting code & software for further information.

Data

Policy information about availability of data

All manuscripts must include a data availability statement. This statement should provide the following information, where applicable:

- Accession codes, unique identifiers, or web links for publicly available datasets
- A description of any restrictions on data availability
- For clinical datasets or third party data, please ensure that the statement adheres to our policy

Data Availability Statement The CRIC data are available from the CRIC Study group upon request and with a Data Use Agreement. Data requests can be made by contacting the CRIC Scientific and Data Coordinating Center at cri-projmgmt@lists.upenn.edu. Data access is controlled due to the terms of the informed consent which does not allow for the data to be posted publicly with open access. The CRIC Study group will typically respond to requests within one week, but the

timeframe for providing data will vary depending on the timeline for completion of a Data Use Agreement between Penn and the receiving institution. Among other standard terms, Data Use Agreements will stipulate that the data only be used for a pre-specified purpose and that the data not be shared with third parties, and that publications of the data must appropriately recognize the source of the data. If at the time of the data request the data are available through a federal repository, the requestor will be referred to the appropriate repository to submit a data request. ARIC data are regularly posted to repositories including dbGaP and BioLINCC in addition, requests for data and verification analysis can be sent to the ARIC data coordinating center at University of North Carolina (aricpub@unc.edu attention David Couper) Source data are provided with this paper. Please see Supplementary Datal Files and Source Data, Figure 2, for minimum datasets that delineate protein associations described herein.

Research involving human participants, their data, or biological material

Policy information about studies with human participants or human data. See also policy information about sex, gender (identity/presentation), and sexual orientation and race, ethnicity and racism.

Reporting on sex and gender	Gender for CRIC participants is reported in Supplemental Table 3 and gender is included in our multivariable analyses of individual proteins. At enrollment, information on participant sex/gender was collected by self-report; there were no sex/gender-based inclusion or exclusion criteria.
Reporting on race, ethnicity, or other socially relevant groupings	Self-reported race of CRIC participants is reported in Supplemental Table 3 and race is included in our multivariable analyses of individual proteins.
Population characteristics	Age, gender, race and other baseline characteristics are described in Supplemental Table 3.
Recruitment	Between 2003 and 2008, the CRIC study enrolled a total of 3,939 ethnically diverse men and women at 7 clinical centers, ages 21 - 74 years, with eGFR 20 - 70 ml/min/1.73 m2 by the simplified Modification of Diet in Renal Disease MDRD equation.14 Eligibility criteria and baseline characteristics of the CRIC cohort have been published. (Feldman et al., 2003) We are not aware of any self-selections bias present among participants that would impact the results of our analysis.
Ethics oversight	The CRIC study protocols adhered to ethics regulations of each institution where participants were enrolled, requiring approval from the following committees: University of Pennsylvania Institutional Review Board, Federalwide Assurance # 00004028; Johns Hopkins Institutional Review Board NA_00044034 / CIR00004697; The University of Maryland, Baltimore Institutional Review Board; University Hospitals Cleveland Medical Center Institutional Review Board; MetroHealth Institutional Review Board; Cleveland Clinic Foundation Institutional Review Board IRB #5969; University of Michigan Medical School Institutional Review Board; Wayne State University Institutional Review Board; University of Illinois at Chicago Institutional Review Board; Tulane Human Research Protection Office, Institutional Review Boards, Biomedical Social Behavioral , IRB #140987; Kaiser Permanente Northern California Institutional Review Board.<br>The CRIC study was approved by the Institutional Review Boards of the participating centers and the research was conducted in accordance with the principles of the Declaration of Helsinki. All study participants provided written informed consent.<br>The Atherosclerosis Risk in Communities (ARIC) Study adhered to ethics regulations from and was approved by a single Institutional Review Board (sIRB) at Johns Hopkins School of Medicine (FWA00005752; IRB00311861) and Institutional Review Boards (IRB) at all participating institutions: University of North Carolina at Chapel Hill, Johns Hopkins University School of Public Health, University of Minnesota, Wake Forest University Health Sciences, University of Mississippi Medical Center, Baylor College of Medicine, University of Texas Houston Health Science Center, and Brigham and Women's Hospital. Study participants provided written informed consent at all study visits.

Note that full information on the approval of the study protocol must also be provided in the manuscript.

Field-specific reporting

Please select the one below that is the best fit for your research. If you are not sure, read the appropriate sections before making your selection.

☒ Life sciences ☐ Behavioural & social sciences ☐ Ecological, evolutionary & environmental sciences

For a reference copy of the document with all sections, see nature.com/documents/nr-reporting-summary-flat.pdf

Life sciences study design

All studies must disclose on these points even when the disclosure is negative.

Sample size	We based our sample size on the number of CRIC participants at Visit 5 (N=3419) with available plasma to be assayed for SomaScan.
Data exclusions	We excluded 53 participants with prevalent kidney failure. Due to the interference of lupus antibodies with aptamers (communication from SomaLogic), we also excluded 12 participants with systemic lupus erythematosus. After 105 samples were excluded that did not pass SomaLogic's quality control standards, the final analytical cohort consisted of 3,249 participants. Fourteen participants were excluded who did not have a baseline measure of eGFR, leaving 3235 individuals eligible for analyses of the primary outcome of a 50% decline in eGFR or kidney failure over 10 years and 3243 participants eligible for analyses for the secondary outcome of a 4-year eGFR decline
Replication	We validated protein - clinical outcome associations in the Atherosclerosis Risk in the Communities study, and we queried three GWAS for genetic validation using Mendelian randomization.
Randomization	This is not application as this was an observational cohort study.
Blinding	The investigators were blinded to group allocation.

Reporting for specific materials, systems and methods

We require information from authors about some types of materials, experimental systems and methods used in many studies. Here, indicate whether each material, system or method listed is relevant to your study. If you are not sure if a list item applies to your research, read the appropriate section before selecting a response.

Materials & experimental systems

n/a	Involved in the study
☒	☐ Antibodies
☒	☐ Eukaryotic cell lines
☒	☐ Palaeontology and archaeology
☒	☐ Animals and other organisms
☒	☐ Clinical data
☒	☐ Dual use research of concern
☒	☐ Plants

Methods

n/a	Involved in the study
☒	☐ ChIP-seq
☒	☐ Flow cytometry
☒	☐ MRI-based neuroimaging
